# Mechanistic Insights into Synergy between Melanin-Targeting Radioimmunotherapy and Immunotherapy in Experimental Melanoma

**DOI:** 10.3390/ijms21228721

**Published:** 2020-11-18

**Authors:** Mackenzie E. Malo, Kevin J. H. Allen, Rubin Jiao, Connor Frank, David Rickles, Ekaterina Dadachova

**Affiliations:** 1 College of Pharmacy and Nutrition, University of Saskatchewan, Saskatoon, SK S7N 5E5, Canada; mackenzie.malo@usask.ca (M.E.M.); kja782@mail.usask.ca (K.J.H.A.); ruj501@mail.usask.ca (R.J.); csf876@mail.usask.ca (C.F.); 2 RadImmune Therapeutics Inc., Burlingame, CA 94010, USA; drickles@radimmune.com

**Keywords:** radioimmunotherapy, DBA/2 mice, anti-PD-1 immunotherapy, lutetium-177, actinium-225

## Abstract

Melanoma incidence continues to rise, and while therapeutic approaches for early stage cases are effective, metastatic melanoma continues to be associated with high mortality. Immune checkpoint blockade (ICB) has demonstrated clinical success with approved drugs in cohorts of patients with metastatic melanoma and targeted radionuclide therapy strategies showed promise in several clinical trials against various cancers including metastatic melanoma. This led our group to investigate the combination of these two treatments which could be potentially offered to patients with metastatic melanoma not responsive to ICB alone. Previously, we have demonstrated that a combination of humanized anti-melanin antibody conjugated to 213Bismuth and anti-PD-1 ICB reduced tumor growth and increased survival in the Cloudman S91 murine melanoma DBA/2 mouse model. In the current study, we sought to improve the tumoricidal effect by using the long-lived radionuclides 177Lutetium and 225Actinium. Male Cloudman S91-bearing DBA/2 mice were treated intraperitoneally with PBS (Sham), unlabeled antibody to melanin, anti-PD-1 ICB, 177Lutetium or 225Actinium RIT, or a combination of ICB and RIT. Treatment with anti-PD-1 alone or low-dose 177Lutetium RIT alone resulted in modest tumor reduction, while their combination significantly reduced tumor growth and increased survival, suggesting synergy. 225Actinium RIT, alone or in combination with ICB, showed no therapeutic benefit, suggesting that the two radionuclides with different energetic properties work in distinct ways. We did not detect an increase in tumor-infiltrating T cells in the tumor microenvironment, which suggests the involvement of alternative mechanisms that improve the effect of combination therapy beyond that observed in the single therapies.

## 1. Introduction

Melanoma, a cancer that forms in the melanocyte cells of the skin where the pigment melanin is produced, continues to be one of the deadliest forms of skin cancer. While the exact etiology of the disease is not clear, it has been established that exposure to ultraviolet radiation significantly increases the risk of developing melanoma [1]. Despite this knowledge, melanoma incidence rates continue to rise, with a projected 100,000 new cases and almost 7000 deaths in the United States in 2020 alone [2]. Surgical resection can provide significant success in early stage melanoma, but metastatic disease is associated with increased morbidity and mortality [3]. The 5 year prognosis of stage III, for example, is extremely variable, ranging from 93% at 5 years (stage IIIA) to 32% at 5 years (stage IIID), with individuals who have metastatic disease at the point of diagnosis making up approximately 4% of cases in 5 year survival rates. New lines of defense must be focused on the treatment and prevention of metastatic disease.

In 2011, the U.S. Food and Drug Administration (FDA) and the European Medicines Agency (EMA) approved the first immune checkpoint blockade (ICB) therapy for treatment of metastatic melanoma. Initially, a monoclonal antibody (mAb) targeting CTLA-4 (ipilimumab) was used to for ICB, followed by mAbs inhibiting PD-1 (nivolumab and pembrolizumab), and combinations of these therapies [4,5]. With the development of these therapies, there was a dramatic decrease in melanoma mortality rates and improved 1 year survival rates, and ICB quickly transitioned into the standard of care [2]. However, the mortality rates for metastatic melanoma are still high, because there are a significant number of cases that do not achieve durable long-term response [6].

The approval of Lutathera^®^ by the FDA and the EMA for the treatment of somatostatin receptor-positive neuroendocrine tumors opened the door for the use of the beta emitter 177Lutetium (^177^Lu) in clinical settings [7]. The recent clinical success of targeted radiation therapy (TRT) with ^177^Lu and the alpha emitter 225Actinium (^225^Ac) against metastatic Castration-Resistant Prostate Cancer demonstrates the specificity, cytotoxic power, and tolerability of TRT [8,9,10].

Our group has shown that melanoma is susceptible to radioimmunotherapy (RIT), a form of TRT. In a phase I clinical trial, we targeted the pigment melanin with a murine IgM 6D2 radiolabeled with the beta emitter 188Rhenium (^188^Re), and demonstrated that the therapy was well tolerated, indicating the potential of melanin as a target [11]. We have since developed a humanized IgG to melanin h8C3, and have found it to be effective in the treatment of B16-F10 murine melanoma in female C57BL/6 mice when radiolabeled with the alpha emitter 213Bismuth (^213^Bi) while not affecting healthy melanin containing tissues [12]. With the success of ICB therapy in the clinical setting of advanced melanoma, we set out to test its effectiveness in combination with our anti-melanin targeted cytotoxic approach of RIT. In our most recent study, we treated Cloudman S91 murine melanoma in the immune-intact DBA/2 (DBA/2NCrl) mouse model with a combination of ^213^Bi-labeled h8C3 mAb and anti-PD-1 ICB [13]. We were able to demonstrate that while ICB or RIT alone only had a modest effect on survival and tumor growth, repeat RIT dosing and the combination of RIT and ICB proved more effective at significantly increasing animal survival and at slowing down tumor growth, while also being well tolerated by the study animals.

Retrospective studies have also suggested that there may be an abscopal effect of external beam radiation and ICB [14,15,16,17]. Based on these observations of synergy and recent successes of TRT, several phase I clinical trials have begun looking specifically at combination treatment using anti-PD-1 inhibitors with TRT in other cancers [18,19].

Encouraged by the effectiveness of the combination of ICB and RIT using the short-lived ^213^Bi radionuclide (46 min physical half-life), in the current study, we have evaluated other radionuclides such as ^177^Lu and ^225^Ac (6.7 and 9.9 days physical half-lives, respectively) to determine the relative effectiveness of RIT targeting melanin with longer-lived beta- and alpha-emitting radionuclides. Additionally, we are interested in understanding the mechanisms of action (MOAs) by which RIT and ICB impart their effect, and whether there is a synergy between these actions that can be further exploited to improve outcomes.

## 2. Results

**Figure 1:**
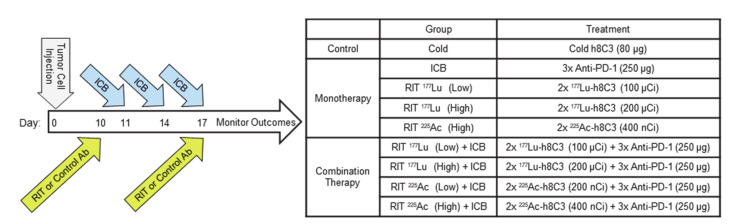
Schematic of the therapy study and cohort descriptions.

In this study, we investigated the effect of anti-PD-1 ICB therapy, or RIT, using the humanized anti-melanin mAb h8C3 conjugated to either ^177^Lu or ^225^Ac, or a combination of these therapies, on tumor growth and survival in the Cloudman S91 murine melanoma in the immune-intact DBA/2 (DBA/2NCrl) mouse model. Male tumor-bearing mice were divided into treatment cohorts that were subjected to a monotherapy or combination therapy following the dosing regimen outlined in Figure 1.

### 2.1. A Combination of ICB with ^177^Lu-h8C3 RIT in Melanoma-Bearing Mice Was More Effective Than Either Therapy Alone

**Figure 2:**
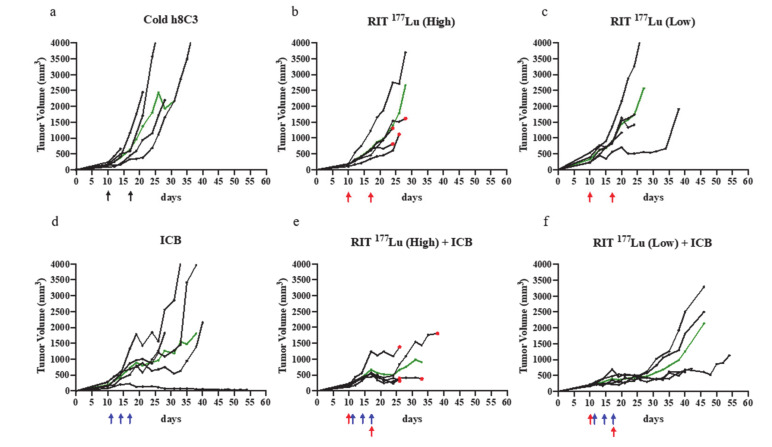
Combination study of anti-PD1 mAb vs. RIT with ^177^Lu-h8C3 mAb. The mice in groups of five were treated with: (**a**) two doses of unlabeled (cold) h8C3 anti-melanin mAb on day 10 and 17 after S91 Cloudman tumor cell inoculation; (**b**) two doses of 200 μCi (High) of ^177^Lu-h8C3 mAb on day 10 and 17 (RIT ^177^Lu High); (**c**) two doses of 100 μCi (Low) of ^177^Lu-h8C3 mAb on day 10 and 17 (RIT ^177^Lu Low); (**d**) three doses of 250 μg anti-PD1 mAb on day 11, 14, and 17 (ICB); (**e**) three doses of 250 μg anti-PD1 mAb on day 11, 14, and 17 with two doses of 200 μCi (High) of ^177^Lu-h8C3 mAb on day 10 and 17 (RIT ^177^Lu High + ICB); (**f**) three doses of 250 μg anti-PD1 mAb on day 11, 14, and 17 with two doses of 100 μCi (Low) of ^177^Lu-h8C3 mAb on day 10 and 17 (RIT ^177^Lu Low + ICB). Day 0 is the day of tumor cell inoculation. Each black tumor volume curve represents a single animal; green line—median tumor volume in the group. Black, blue, and red arrows indicate the injection of cold antibody, anti-PD1 antibody, and RIT, respectively. Red dots indicate the death of the animal from radiotoxicity.

In this study, the combination of anti-PD-1 ICB treatment and RIT utilizing the beta emitter ^177^Lu radiolabeled h8C3 showed a significant reduction in tumor doubling time, and notable effect on overall survival when compared to any monotherapy (Table 1). Figure 2 shows the tumor volume change in response to monotherapy (^177^Lu RIT alone, anti-PD-1 ICB alone) or combination therapy (^177^Lu RIT + ICB). Unlabeled (“cold”) h8C3 mAb was utilized as control. Two doses of 200 µCi (high) or 100 µCi (low) ^177^Lu-h8C3 or unlabeled h8C3 were administered on days 10 and 17 (Figure 2a–c).

**Figure 3:**
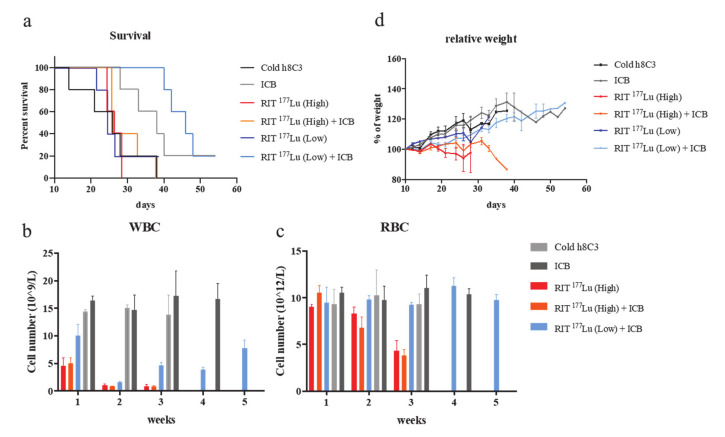
Survival of DBA/2 mice bearing S91 Cloudman tumor cells treated with anti-PD1 mAb and/or RIT with ^177^Lu-h8C3 mAb and toxicity evaluation. (**a**) Survival. Unlabeled h8C3 antibody (cold), ICB, high/low doses of RIT ^177^Lu with/without ICB treatment; (**b**) WBC count in RIT ^177^Lu with/without ICB treatment; (**c**) RBC count in RIT ^177^Lu with/without ICB treatment; (**d**) relative body weight. Percentage of the body weight was calculated based on each animal’s weight at day 10 when the first treatment was administered.

Administration of 100 µCi (low-dose) ^177^Lu RIT (Figure 2c) resulted in modest, although not significant, suppression of tumor growth (*p* = 0.059) when compared to cold h8C3 (Figure 2a), and no difference was observed between the low and high (200 µCi) ^177^Lu RIT monotherapy doses (*p* = 0.724) (Figure 2b,c). ICB alone (Figure 2d) had modest effect on tumor growth that attained statistical significance (*p* = 0.033), with 3 doses of 250 µg anti-PD1 mAb on days 11, 14, and 17 showing a varied response, from complete cure to no effect when compared to unlabeled h8C3 (Figure 2a,d). The ICB monotherapy regimen also resulted in improved overall survival (Figure 3a, Table 1).

The combination of ^177^Lu RIT (low) with ICB (Figure 2f) resulted in significant reduction in the rate of tumor growth, as determined by calculating tumor volume doubling time (T_d_), when compared to the cold h8C3 control group (*p* = 0.004), to the ^177^Lu RIT (low) monotherapy (*p* = 0.027), and to the ICB monotherapy (*p* = 0.047); in addition to demonstrating prolonged median survival ranging from 8 to 20 days (Table 1, Figure 3a). In contrast, neither ^177^Lu RIT (high) monotherapy (Figure 2b), or the combination of ^177^Lu RIT (high) with ICB treatment (Figure 2e) resulted in any significant decrease in tumor growth, or extension of survival (Table 1, Figure 3a). In terms of toxicity, low-dose ^177^Lu RIT in combination with ICB was well tolerated with minimal hematologic toxicity upon 5 weeks of treatment. (Figure 3b,c) and the maintenance of body weight (Figure 3d), whereas the 2 doses of 200 µCi of ^177^Lu RIT monotherapy or in combination with ICB reached the maximum tolerated dose as indicated by extreme weight loss (Figure 3d) and decreased white blood cell (WBC) and red blood cell (RBC) counts that did not recover (Figure 3b,c).

**Figure 4:**
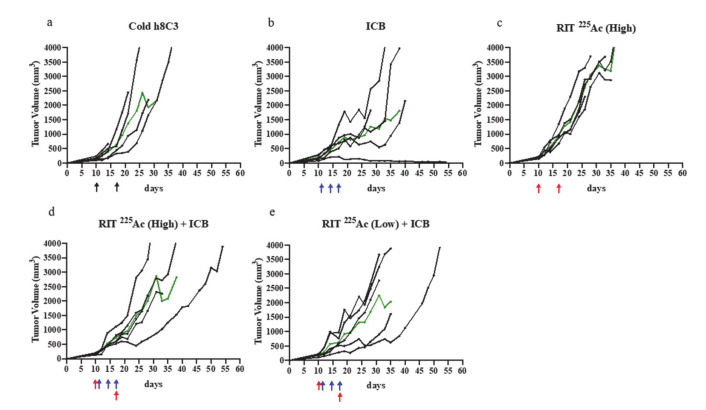
Combination study of anti-PD1 mAb vs. RIT with ^225^Ac-h8C3 mAb. The mice in groups of five were treated with: (**a**) two doses of unlabeled (cold) h8C3 anti-melanin mAb on day 10 and 17 after S91 Cloudman tumor cell inoculation; (**b**) three doses of 250 μg anti-PD1 mAb on day 11, 14, and 17 (ICB); (**c**) two doses of 200 nCi (High) of ^225^Ac-h8C3 mAb on day 10 and 17 (RIT ^255^Ac High); (**d**) three doses of 250μg anti-PD1 mAb on day 11, 14, and 17 with two doses of 200 nCi (High) of ^225^Ac-h8C3 mAb on day 10 and 17 (RIT ^255^Ac High + ICB); (**e**) three doses of 250 μg anti-PD1 mAb on day 11, 14, and 17 with two doses of 100nCi (Low) of ^225^Ac-h8C3 mAb on day 10 and 17 (RIT ^255^Ac Low + ICB). Day 0 is the day of tumor cell inoculation. Each black tumor volume curve represents a single animal, green line—median tumor volume in the group. Black, blue, and red arrows indicate the injection of cold antibody, anti-PD1 antibody, and RIT, respectively.

**Figure 5:**
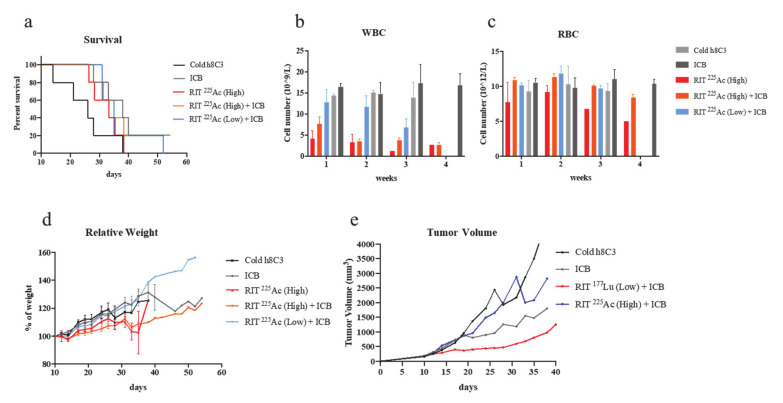
Survival of DBA/2 mice bearing S91 Cloudman tumor cells treated with anti-PD1 mAb and/or RIT with ^255^Ac-h8C3 mAb and toxicity evaluation. (**a**) Survival. Unlabeled h8C3 antibody (cold), ICB, high/low doses of RIT ^255^Ac with/without ICB treatment. (**b**) WBC count in RIT ^255^Ac with/without ICB treatment. (**c**) RBC count in RIT ^177^Lu with/without ICB treatment. (**d**) Relative body weight. Percentage of the body weight was calculated based on each animal’s weight at day 10 when the first treatment was administered. (**e**) Median tumor volume curves for monotherapy and combination studies.

Assessment of the effectiveness of ^225^Ac-labelled h8C3 in combination with anti-PD-1 ICB treatment in Cloudman S91 murine melanoma model did not result in any significant therapeutic effect (Figure 4a–e). When comparing tumor doubling time T_d_ or median survival of ^225^Ac-h8C3 RIT (high-dose) monotherapy (Figure 4c) to cold h8C3, there was a modest, but not significant effect—*p* = 0.0502 (Table 1). Combining ^225^Ac-h8C3 RIT with ICB therapy appeared to negate the tumor-suppressive effect of the ICB monotherapy, resulting in no change in T_d_ and not significant reduction in survival (Table 1, Figure 3d,e, Figure 5a). The two doses of 400 nCi (high) and 200 nCi (low) ^225^Ac-h8C3 on days 10 and 17 alone, or in combination ICB therapy were well tolerated in terms of WBC and RBC counts (Figure 5b,c) and body weight (Figure 5d). Figure 5e provides the comparison between the median tumor volume in monotherapies and combination therapy groups.

### 2.2. Immunohistochemistry Detected More Necrosis in Combination-Treated Tumors and No Difference in CD3+ Cells and Ki67 Positivity

**Figure 6:**
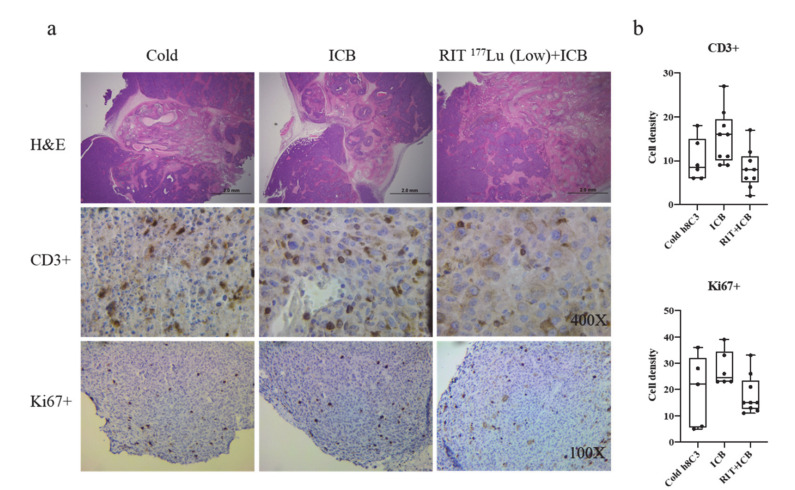
H&E staining and IHC staining of tumors. Panel (**a**): First row, H&E staining of tumors from cold h8C3, ICB, and RIT ^177^Lu (low) + ICB treatment groups. Second row, anti-CD3 IHC staining under 400X magnification; brown staining surrounds the nuclear indicate the CD3+ cells. Bottom row, anti-Ki67 IHC staining under 100X magnification, and brown staining overlaps with the nuclear indicate the Ki67+ cells. Panel (**b**): upper row—number of CD3-positive cells in cold h8C3, ICB, and RIT + ICB treatment group; lower row—number of Ki67-positive cells in cold h8C3, ICB, and RIT + ICB treatment group.

When we completed the therapy, which consisted of treatment with a combination of ICB and/or RIT, mice that had survived to the end point were sacrificed and tumors were assessed histologically. Tumors from the surviving mice in the cold h8C3, ICB, and RIT ^177^Lu (Low) + ICB groups were evaluated for percentage of necrosis, CD3+ cells and Ki67-positive cells. Analysis of H&E-stained slides showed that, on average, 30%, 26.7%, and 55% tumor necrosis was observed in the cold h8C3, ICB, and RIT ^177^Lu (Low) + ICB groups, respectively (Figure 6a, upper row). CD3+ cells represent the infiltrating T cells in the tumor. An average of 10–15 CD3+ cells were observed from each view (Figure 6a, middle row), making no significant difference between the groups (Figure 6b, upper row). To investigate the number of proliferating cells, anti-Ki67 antibody was used to stain the tumor sections (Figure 6a, lower row). We did not detect any significant difference in the number of Ki67-positive cells when comparing each treatment group (Figure 6b, lower row).

### 2.3. Mechanistic Studies Showed No Difference in Tumor-Infiltrating T Cells Between the Treatment Groups

**Figure 7:**
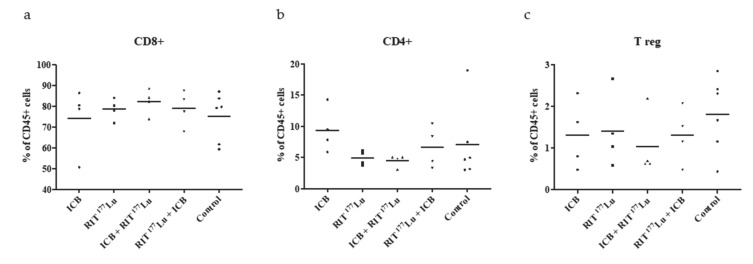
Flow cytometry data for determination of tumor-infiltrating T cells for possible synergistic effects between anti-PD1 and ^177^Lu-h8C3 antibody. Groups of four tumor-bearing mice (three treatments of anti-PD1), single injection of ^177^Lu, three treatments of anti-PD1 followed by one treatment of ^177^Lu-8hC3, and one treatment of ^177^Lu-h8C3) or six mice (control) were treated and sacrificed on day 20. Tumors were harvested and analyzed by (**a**) comparing % of CD8+ cells among CD45+ cells, (**b**) comparing % of CD4+ cells among CD45+ cells, and (**c**) comparing % of Treg+ cells among CD45+/CD4+/foxP3+ cells for each cohort. Each data point represents one animal in each group. No significance was found in any treatment group relative to control.

To investigate the role of tumor-infiltrating T cells on the tumor growth suppression observed in the ^177^Lu RIT and immunotherapy cohorts, we performed a flow cytometry study. Male DBA/2 mice bearing S91 Cloudman tumors were treated with: 1) anti-PD1 alone, 2) a single treatment of low-dose ^177^Lu RIT, 3) three treatments of anti-PD1 followed by low-dose ^177^Lu RIT, or 4) ^177^Lu RIT followed by three treatments of anti-PD1. These were all compared to an untreated control group. Tumors were harvested 20 days post tumor inoculation and analyzed by comparing the percentage of CD8+ and CD4+ cells in the CD45+ cell population (Figure 7a,b), as well as the percentage of T_reg_+ cells in the CD45+/CD4+/foxP3+ cell population (Figure 7c). While RIT and ICB combination therapy imparted a significant effect on tumor growth and overall survival in our therapeutic study, we did not observe any significant differences in the presence of tumor infiltrating T cells between any of the cohorts and the untreated control, or between cohorts themselves.

## 3. Discussion

In this study, we utilized male DBA/2 mice bearing Cloudman S91 tumors, a syngeneic melanoma model [20], to assess the combination of immunotherapy with long-lived alpha- and beta-emitting RIT. We and others have confirmed the utility of this model in evaluating the effectiveness of immunotherapy alone or in combination with other treatments and have demonstrated anti-PD-1 provides modest tumor control and improved survival [13,21,22]. Additionally, we have shown that the combination of anti-PD-1 therapy with RIT using ^213^Bi-labeled h8C3, a humanized mAb targeting melanin, slows down tumor growth by 1.5 fold times and improves survival in this model [13].

Working in an immunocompetent model with a syngeneic tumor allows us to evaluate the interaction between the cancer and the activated immune cells, which is of particular importance as our goal with this study was to investigate the role of activated immune cells in the tumor microenvironment (TME) while improving the effectiveness of our RIT by utilizing alternative to ^213^Bi radionuclides. We selected ^177^Lu (6.7 d half-life) and ^225^Ac (9.9 d half-life) to compare the therapeutic utility of beta- versus alpha-emitting isotopes in treating melanoma, as both are relatively long-lived, allowing for comparison, and both are clinically relevant isotopes [8,9,10,18,19]. Alpha particle radiation has a higher linear energy transfer (LET) resulting in increased density of ionizing events along the particle track with a shorter range of penetration (40–100 µm), whereas beta emission is less densely ionizing with a longer range (200–1200 µm) [23]. To compensate for the higher cytotoxic capacity of ^225^Ac relative to ^177^Lu, it was necessary to use different doses for each isotope to achieve similar relative biological effectiveness. Directly comparing the two isotopes in non-melanoma systems found that comparative cytotoxic effect was achieved when using ^177^Lu at a 100–700-fold higher dose [24,25].

We observed that RIT alone, using either ^177^Lu or ^225^Ac, did not control tumor growth (Table 1, Figure 2 and Figure 4). This contrasts with our previous studies, where we assessed the cytotoxic effect of the shorter-lived alpha emitter, ^213^Bi [12,13]. In those works, we found that both the S91 Cloudman model and the more rapidly dividing B16F10 model were highly susceptible to RIT with ^213^Bi. This suggests that RIT alone is most effective in treating melanoma when a high-energy cytotoxic dose is delivered at a high dose rate, as ^213^Bi has this capacity owing to its 46 min half-life. In using 231Bi, we are delivering two high-energy alpha particles per 46 min decay period, whereas, with 225Ac, we can deliver a larger dose, five alpha particles, but over a 10 day decay period, or we deliver an even lower energy dose from beta particles with 177Lu, again over a long decay period (6.7 days). While 213Bi generates less energy per decay, it will go through many more decay events than the long-lived isotopes over a given amount of time, and therefore will deposit a larger dose in a short period of time. This demonstrates that when selecting an isotope, factors such as total dose, dose rate, and particle range must be considered. We have shown, in in vitro killing assays comparing the three isotopes, that only ^213^Bi shows any cytotoxic effect at day 3 post dosing, whereas ^177^Lu and ^225^Ac produce equitoxic effects at 7 days post treatment [24]. The longer half-life of ^177^Lu and ^225^Ac may have resulted in a missed window of opportunity to slow tumor growth in this model.

While RIT with either ^177^Lu and ^225^Ac isotope alone provided little therapeutic advantage, a profound anti-tumor effect was achieved, and improved survival was observed when low-dose ^177^Lu RIT was combined with anti-PD-1 ICB therapy (Table 1). Concurrent ICB therapy with the low dose of ^177^Lu RIT delayed exponential tumor growth until day 30, whereas exponential growth was observed in the cold h8C3 mAb group and in the anti-PD-1 group by day 10 (Figure 2 and Figure 5e). This delay in tumor growth resulted in enhanced overall survival (Table 1, Figure 3a). The combination of low-dose ^177^Lu RIT with ICB resulted in an obvious therapeutic advantage, suggesting that either the two approaches are producing a synergistic anti-tumor immunity effect whose mechanism is not reflected in tumor infiltrating T cells subpopulation changes, as measured by our methodology, or that the effect of ICB delayed tumor growth enough for ^177^Lu to more effectively deposit its cytotoxic dose, thereby resulting in an overall improvement in tumor response. The lack of significant benefit (synergy) of combining high-dose ^177^Lu RIT with ICB could be due to the off target systemic immuno-suppressive effects of high-dose ^177^Lu RIT, such as bone marrow toxicity, as humanized and human antibodies have been shown to bind strongly to murine FcRn receptors, potentially resulting in bone marrow irradiation [26].

Interestingly, while combination therapy was effective with ^177^Lu, we found that this was not the case when using ^225^Ac. The physical characteristics of alpha and beta radiation result in different biological responses. To compare, we selected isotopes with similar decay times, and we attempted to administer similar equitoxic doses. Despite these considerations, we found ^177^Lu to be effective in combination with ICB, while ^225^Ac was not. The higher LET and shorter range of ^225^Ac possibly resulted in more direct killing of cells by DNA double-strand breaks (DSBs), whereas the lower LET and longer range of ^177^Lu resulted in DNA damage and generation of reactive oxygen species (ROS) in a wider swath. This could result in differences to the bystander effect caused by radiation, whereby radiation events in irradiated cells and tissue initiate a response in surrounding cells and tissue [27]. The difference in effect of the alpha- and beta-emitting radiation in this study suggests that the “window of opportunity” provided by ICB therapy for the RIT to deposit its dose might not be the only piece of the puzzle. The suggested synergy observed in the ^177^Lu RIT combination therapy could also be due to the way in which beta-emitting radiation interacts with the tumor and the TME.

Radiation therapy traditionally has been utilized for its cytotoxic effect on tumors, wherein it kills by delivering a lethal dose of energy to its target; but radiation can also have a systemic effect that appears to be mediated via the immune system. This is well summarized in a review by Lumnisczky et al. [28]. Ultimately, radiation-targeted tumor cells have increased immunogenicity and produce immune-stimulatory cytokine and chemokines, antigen-presenting cells (APCs) have increased access to antigens in the TME, and T cell activation and tumor infiltration increases. The observed relationship between radiation and the immune system makes the combination of these therapies of interest in a landscape where ICB is the standard of care.

To expand ICB therapy efficacy it is necessary to first ensure tumor infiltration by activated T cells. For example, tumor suppression by blocking PD1 checkpoints is only effective if there are CD8+ T cells that are negatively regulated by PD-1/PD-L1-mediated adaptive immune resistance [29]. PD-1 ligand overexpression in response to inflammation can create this target for ICB therapy [5]. Inflammation can result in improved tumor immunogenicity that can sensitize previously non-ICB-responsive tumors to ICB [30]. Several recent studies have combined the power of ^177^Lu-targeted radionuclide therapy with ICB therapy in mouse models of cancer [31,32]. Both groups demonstrated a significant increase in survival with combination beyond that observed in single therapies. By combining beta- or alpha-emitting long-lived isotope RIT with ICB, we aimed to provide a targeted cytotoxic dose to the tumor, increase inflammation in the TME, and initiate an immune response. Following this initial attack, we intended to then disable the immune checkpoints with ICB therapy and fortify the tumoricidal effect of RIT. While we did see this as predicted in our therapeutic study (Figure 5e, Table 1), we did not observe a correlating increase in tumor infiltrating T cells in the TME in our fluorescence-activated cell sorting (FACS) study (Figure 7).

A synergistic effect has been demonstrated between external beam radiation therapy (RT) administered in combination with ICB therapy in animal experiments, pilot studies, and clinical trials. Two independent groups presented cases where concurrent CTLA-4 ICB and RT resulted in regression at both the targeted and non-targeted melanoma lesions, demonstrating an abscopal effect [33,34]. A subsequent phase I clinical trial assessed the systemic anti-melanoma immune response in stage IV melanoma patients administered ipilimumab and external beam RT via an abscopal effect and found a benefit to 55% of patients [35]. In a study by Park et al., using the B16-OVA melanoma mouse model, they were able to show that stereotactic ablative radiotherapy induced an abscopal effect, and this effect was enhanced by the use of anti-PD-1 antibodies [16]. In each of these studies, there was a correlation between anti-tumor effect of RT and ICB and increases in activated T cell populations. More recently, Rouanet et al. presented a study in which they clearly demonstrate that TRT targeting melanin in the murine B16F10 melanoma model caused immunogenic death and resulted in recruitment of adaptive and innate immune cells to the TME [36]. Furthermore, they demonstrated that tumor suppression and prolonged survival was achieved when TRT was administered in concert with ICB, adding support to the coordinated roles of TRT and ICB in affecting tumor suppression in the context of the immune mechanism.

Considering the weight of studies demonstrating the correlation between increases in tumor infiltrating T cells in the TME and efficacy of radiation and ICB combination therapy, we decided that a complete histological study on tumors collected when therapeutic cohorts reached their defined end point could provide valuable information. H&E staining showed that there was a higher degree of tumor necrosis when RIT was combined with ICB treatment. To visualize the tumor infiltrating T cells, we stained the tumors with anti-CD3 antibody, which binds all T cells. There was no statistical difference in the CD3+ cell numbers observed in the RIT combined with the ICB group, compared to cold antibody control and ICB treatment alone, agreeing with what was observed in the FACS analysis. The presence of Ki67 was used as an indication of cell proliferation. We found that the center of the tumor had almost no Ki67 staining, with the majority of the positive cells present on the outer area of the tumor. This could be due to the cell death in the center, compared to the newly growing tumor. The averages of Ki67+ cells in cold antibody control and ICB alone treatments were higher, although not significantly, than that in the combined therapy, indicating the faster growth of the tumor. This is consistent with the decreased tumor growth rate in the combination therapy group (Table 1).

In conclusion, in the current study, we were able to successfully identify ^177^Lu as an isotope well suited for the treatment of the murine melanoma cell line Cloudman S91 in the immune-intact DBA/2 (DBA/2NCrl) mouse model using a humanized mAb targeting the pigment melanin. Furthermore, the combination of ^177^Lu RIT with ICB via the anti-PD-1 antibody proved to further enhance the tumoricidal effect of both therapies suggesting a synergistic effect. While other groups have been effective at correlating an increase in tumor infiltrating T cells in the TME with the abscopal effect of RT and ICB, we did not observe this in this study. This could suggest that the presence of tumor infiltrating T cells is a temporal event that can only be captured in a narrow timeframe, or that other mechanisms are also at play that create the suggested synergistic effect we observed. Future studies will be directed at identifying the MOA that imparts this suggested synergy.

## 4. Materials and Methods

### 4.1. Antibodies, Reagents, and Radionuclides 

Rat IgG2a to mouse PD-1 (Programmed death-1) also known as CD279 was acquired from Bio X Cell (West Lebanon, NH, USA). Aragen Bioscience (Morgan Hill, CA, USA) manufactured the humanized melanin-binding 8C3 mAb (h8C3). ^225^Ac was purchased from Oak Ridge National Laboratory (Oak Ridge, TN, USA). ^177^Lu was purchased from McMasters University (Hamilton, ON, Canada). Bifunctional chelating agent (BCA) S-2-(4-Isothiocyanatobenzyl)-1,4,7,10-tetraazacycododecane tetraacetic acid (DOTA) was synthesized by Macrocyclics (Dallas, TX, USA). Fluorescent conjugated mouse antibodies (anti-CD45+, anti-CD4+, anti-foxP3) were procured from ThermoFisher (Waltham, MA, USA).

### 4.2. Conjugation to BCA DOTA and Radiolabeling of h8C3 mAb 

DOTA conjugation to h8C3 was performed in a similar manner to our previous work with this antibody with a minor alteration [13]. A 10-fold molar excess of DOTA-SCN was used in place of a 10-fold excess of CHXA’’-SCN. ^225^Ac labeling of DOTA-conjugated h8C3 was performed in an analogous method to that in Dawicki et al. [37]. All radiolabeling yields were greater than 99%. ^177^Lu labeling was performed in a similar manner using a 10:1 µCi: µg ratio of isotope to DOTA conjugated mAb.

### 4.3. Murine Cloudman S91 Melanoma Model

All animal studies were approved by the Animal Research Ethics Board of the University of Saskatchewan (Animal use protocol #20170006, approved on 28/02/2019). DBA/2 male mice (Charles River Laboratories, Sherbrooke, Canada) were purchased and treated as previously described [13]. In brief, 3 million Cloudman S91 cells were injected subcutaneously into the right flank of the mice.

### 4.4. Combination Treatment with Anti-PD-1 Immunotherapy and RIT 

Male DBA/2 mice bearing Cloudman S91 with approximately 150 mm^3^ sized tumors were randomized into the groups of five and treated with either: a dose of 80 µg unlabeled (cold) h8C3 on day 10 and 17 after tumor cell inoculation; three doses of 250 µg anti-PD-1 mAb on days 11, 14, and 17 as in Curran et al. [38]; two doses of RIT on day 10 and 17; three doses of anti-PD-1 mAb on days 11, 14, and 17 and two dose of RIT on day 10 and 17. Two different isotopes, ^177^Lu and ^225^Ac, were used as a low and a high dose. The RIT doses were as follows: 100 µCi ^177^Lu-h8C3 (Low) and 200 µCi ^177^Lu-h8C3 (High) and 200 nCi ^225^Ac-h8C3 (Low) and 400 nCi ^225^Ac-h8C3 (High). The full treatment timeline is shown in Figure 1. The weight of the mice was measured every three days and the blood samples were collected and examined weekly for WBC and RBC counts. The mice were humanely sacrificed as described in the animal use protocol when they reached the Humane Intervention Point (HIP) defined in our animal protocol.

### 4.5. Immunohistochemistry 

At the completion of the therapy study surviving mice were humanely sacrificed, and tumors were collected and fixed in 10% formalin. Fixed tumors were embedded in paraffin and 5μm section were prepared for hematoxylin and eosin (H&E) or immunohistochemistry (IHC) staining. Anti-CD3 (1:700 dilution, Ab16669, Abcam, Cambridge, UK) and anti-Ki67 (1:200 dilution, Ab16667, Abcam, Cambridge, UK) antibodies were used for IHC as previously described [37]. Images were taken using a Labomed Lx400 microscope with a Motic Moticam S2 camera. For CD3+ staining, three selected views under 400× magnification from each of 3 tumors were counted, for a total of 9 section views for each treatment group. To investigate the number of proliferating cells, anti-Ki67 antibody was used to stain the tumor sections. As there were few Ki67-positive cells, a lower magnification (100×) was used.

### 4.6. Mechanism of Action Studies 

Male DBA/2 mice bearing Cloudman S91 tumors, approximately 150 mm^3^ in size, were randomized into the groups of five and treated with: one control treatment of PBS on day 10; one injection of 100 µCi ^117^Lu-h8C3 on day 10; three injections of 250 µg anti-PD-1 mAb on days 11, 14, and 17; one injection of 100 µCi ^117^Lu-h8C3 on day 10 and three injections of 250 µg anti-PD-1 mAb on days 11, 14, and 17; three injections of 250 µg anti-PD-1 mAb on days 11, 14, and 17 and one injection of 100 µCi ^177^Lu-h8C3 on day 17 (Figure 1). Tumors were harvested from all mice on day 20, then immediately dissociated using a mouse tumor dissociation kit (Milteny Biotec, Bergisch Gladbach, Germany) and gentleMACs dissociator (Milteny Biotec, Bergisch Gladbach, Germany) following manufacturers protocols. FACS was completed on a CytoFLEX cytometer (Beckman Coulter, Brea, CA, USA) to evaluate for the presence of the tumor infiltrating T cells in the tumors. For that, the cells were washed then incubated with a mixture of anti-mouse fluorescent conjugated antibodies. FACS results were analyzed using FlowJo software (Tree Star Inc., Ashland, OR, USA) and immune cells were identified by gating on CD45+ cells. CD8+ T cells were identified as CD45^+^/CD8^+^. CD4+ were identified as CD45^+^/CD4^+^. Treg cells were identified as CD45^+^/CD4^+^/foxP3^+^. Appropriate fluorescence minus one (FMO) controls were collected to confirm fluorescent shifts.

### 4.7. Statistics 

Statistical analysis was conducted using Prism8 software (GraphPad, San Diego, CA, USA). A two-tailed *t*-test was performed to compare tumor doubling times between treatments. A logrank test reporting chi-square values was performed to compare differences in survival between treatments. A *p*-value < 0.05 was considered statistically significant. 

Tumor doubling time (*T_d_*) was calculated as:

(1) $$T_{d}=\frac{ln\left(2\right)}{r}$$

Whereas specific growth rate (r) was determined by:

(2) $$r=\frac{ln\left(\frac{V_{x}}{V_{1}}\right)}{\left(t_{x}-t_{1}\right)}$$

*V_x_* refers to the tumor volume at day *x*, and *t* is time measured in days [39]. A Kaplan–Meier survival curve with the median survival (days) was generated using GraphPad Prism8.

## Figures and Tables

**Table 1:** Tumor doubling time (T_d_) and median survival.

Treatment Group	*T_d_* (Days) Mean ± SEM	Student T-Test *p* Value	Median Survival (Days)	Log-Rank Test Chi Square/*p* Value
Cold	3.7 ± 0.5	0.033 *	26	3.918
ICB	6.8 ± 1.2		38	0.048 *
Cold	3.7 ± 0.5	0.059 ns	26	0.078
RIT ^177^Lu (Low)	6.6 ± 1.2		24	0.780 ns
Cold h8C3	3.7 ± 0.5	0.071 ns	26	0.024
RIT ^177^Lu (High)	5.0 ± 0.4		26	0.876 ns
Cold	3.7 ± 0.5	0.004 *	26	9.701
RIT ^177^Lu (Low) + ICB	14.7 ± 2.7		46	0.0018 *
Cold	3.7 ± 0.5	0.0683 ns	26	0.347
RIT ^177^Lu (High) + ICB	16.0 ± 5.9		26	0.556 ns
ICB	6.8 ± 1.2	0.047 *	38	1.071
RIT ^177^Lu (Low) + ICB	14.7 ± 2.7		46	0.301 ns
ICB	6.8 ± 1.2	0.212 ns	38	3.010
RIT ^177^Lu (High) + ICB	16.0 ± 5.9		26	0.083 ns
RIT ^177^Lu (Low)	6.6 ± 1.2	0.027 *	24	6.102
RIT ^177^Lu (Low) + ICB	14.7 ± 2.7		46	0.014 *
RIT ^177^Lu (High)	5.0 ± 0.4	0.097 ns	26	1.713
RIT ^177^Lu (High) + ICB	16.0 ± 5.9		26	0.1906
Cold	3.7 ± 0.5	0.0502 ns	26	0.670
RIT ^225^Ac (High)	5.1 ± 0.4		33	0.413 ns
Cold	3.7 ± 0.5	0.048 *	26	2.037
RIT ^225^Ac (High) + ICB	6.2 ± 0.9		33	0.154 ns
ICB	6.8 ± 1.2	0.787 ns	38	0.387
RIT ^225^Ac (Low) + ICB	6.4 ± 0.8		35	0.534 ns
ICB	6.8 ± 1.2	0.667 ns	38	0.184
RIT ^225^Ac (High) + ICB	6.2 ± 0.9		33	0.668 ns
RIT ^225^Ac (High)	5.1 ± 0.4	0.339 ns	33	0.452
RIT ^225^Ac (High) + ICB	6.2 ± 0.9		33	0.501 ns
RIT ^177^Lu (Low) + ICB	14.7 ± 2.7	0.018 *	46	1.270
RIT ^225^Ac (High) + ICB	6.2 ± 0.9		33	0.260 ns

* statistically significant *p* value; ns—not significant.

## Abbreviations

ICB	Immune checkpoint blockade
mAb	Monoclonal antibody
TRT	Targeted radiation therapy
RIT	Radioimmunotherapy
WBC	White blood cell
RBC	Red blood cell
TME	Tumor microenvironment
LET	Linear energy transfer
FACS	Fluorescence-activated cell sorting
